# 4-[(4-Chloro­phen­yl)diazen­yl]-3-meth­oxy­aniline

**DOI:** 10.1107/S160053681102472X

**Published:** 2011-06-30

**Authors:** Mohammad Kazem Rofouei, Zahra Ghalami, Jafar Attar Gharamaleki, Giuseppe Bruno, Hadi Amiri Rudbari

**Affiliations:** a Faculty of Chemistry, Tarbiat Moallem University, Tehran, Iran; b Dipartimento di Chimica Inorganica, Universita di Messina, Messina, Italy

## Abstract

The title compound, C_13_H_12_ClN_3_O, exhibits a *trans* geometry about the N=N double bond in the solid state. The dihedral angle between the rings is 22.20 (8)°. Inter­molecular N—H⋯O hydrogen bonds between the amine and meth­oxy groups lead to the formation of a chain-like polymer along the *c* axis with a *C*(6) graph set. There is also weak non-classical C—H⋯N hydrogen bonds involving an aromatic C—H group and a diazenyl N atom, which connect the chains into a two-dimensional framework.

## Related literature

For applications of diazo­nium compounds, see: Patai (1978 ▶); Hunger *et al.* (2005 ▶). For the synthesis and crystal structures of Hg(II) and Cd(II) complexes with [1,3-bis­(2-meth­oxy­phen­yl)]triazene, see: Rofouei, Hematyar *et al.* (2009 ▶); Rofouei, Melardi *et al.* (2009 ▶).
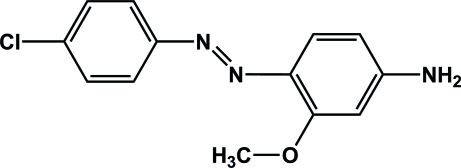


## Experimental

### 

#### Crystal data

C_13_H_12_ClN_3_O
*M* *_r_* = 261.71
Monoclinic, 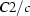

*a* = 15.398 (2) Å
*b* = 12.132 (2) Å
*c* = 14.276 (2) Å
β = 107.65 (1)°
*V* = 2541.3 (7) Å^3^
*Z* = 8
Mo *K*α radiation
μ = 0.29 mm^−1^
*T* = 293 K
0.30 × 0.17 × 0.11 mm

#### Data collection

Bruker APEXII CCD diffractometer
Absorption correction: multi-scan (*SADABS*; Bruker, 2005 ▶) *T*_min_ = 0.698, *T*_max_ = 0.746
42735 measured reflections
2778 independent reflections
2481 reflections with *I* > 2σ(*I*)
*R*_int_ = 0.019

#### Refinement

*R*[*F* ^2^ > 2σ(*F* ^2^)] = 0.039
*wR*(*F* ^2^) = 0.118
*S* = 1.04
2778 reflections
172 parameters
H atoms treated by a mixture of independent and constrained refinement
Δρ_max_ = 0.37 e Å^−3^
Δρ_min_ = −0.39 e Å^−3^

### 

Data collection: *APEX2* (Bruker, 2005 ▶); cell refinement: *SAINT-Plus* (Bruker, 2005 ▶); data reduction: *SAINT-Plus*; program(s) used to solve structure: *SHELXS97* (Sheldrick, 2008 ▶); program(s) used to refine structure: *SHELXL97* (Sheldrick, 2008 ▶); molecular graphics: *SHELXTL* (Sheldrick, 2008 ▶); software used to prepare material for publication: *SHELXTL*.

## Supplementary Material

Crystal structure: contains datablock(s) I, global. DOI: 10.1107/S160053681102472X/bh2358sup1.cif

Structure factors: contains datablock(s) I. DOI: 10.1107/S160053681102472X/bh2358Isup2.hkl

Supplementary material file. DOI: 10.1107/S160053681102472X/bh2358Isup3.cml

Additional supplementary materials:  crystallographic information; 3D view; checkCIF report

## Figures and Tables

**Table 1:** Hydrogen-bond geometry (Å, °)

*D*—H⋯*A*	*D*—H	H⋯*A*	*D*⋯*A*	*D*—H⋯*A*
N1—H1*A*⋯O^i^	0.85 (2)	2.47 (2)	3.222 (2)	147 (2)
C12—H12⋯N3^ii^	0.93	2.62	3.379 (2)	140

Symmetry codes: (i) 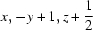; (ii) 
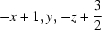
.

**Fig. 1.**
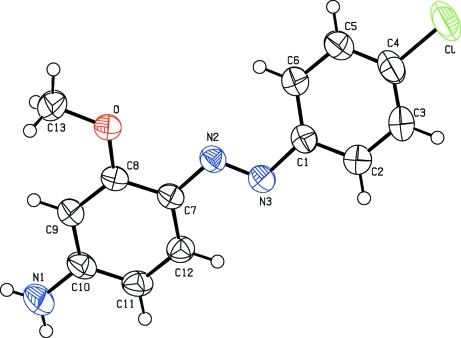
The molecular structure of the title compound, displacement ellipsoids are drawn at 50% probability level.

**Fig. 2.**
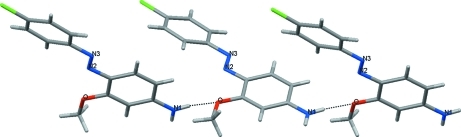
N1—H1A···O hydrogen bonds between molecules, to produce chain-like polymers along the c crystallographic axis.

**Fig. 3.**
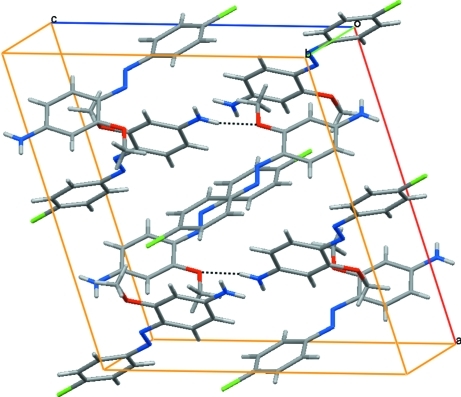
The crystal packing diagram of the title compound.

## supplementary crystallographic information

### Comment

Diazonium ions are present in solutions such as benzenediazonium chloride solution. They contain an -N_2_^+^ group. For example in the case of benzenediazonium chloride, this is attached to a benzene ring. The most important application area of these compounds is organic synthesis of azo dyes (Patai, 1978; Hunger *et al.*, 2005). Diazenyl compounds characterized by having a diazo group (—N═N—) commonly adopt the *trans* configuration in the ground state. We have previously reported the synthesis of Hg(II) and Cd(II) complexes with [1,3-bis(2-methoxyphenyl)]triazene (Rofouei, Hematyar, Ghoulipour & Gharamaleki, 2009; Rofouei, Melardi, Khalili, Ghaydari & Barkhi, 2009).

When attempting to prepare an asymmetric triazene compound using *p*-chloroaniline and *m*-anizidine, we instead obtained the title diazo compound, C_13_H_12_ClN_3_O (Fig. 1). The molecule adopts the *trans* configuration and the C1—N3—N2—C7 dihedral angle is 175.50 (10)°. The C10—N1, C7—N2 and C1—N3 bond lengths are 1.3657 (19), 1.3985 (16) and 1.4206 (17) Å, respectively, consistent with single and double bonds between related C and N atoms. In the crystal lattice of the title compound, the molecules are linked into chain-like polymers along the *c* crystallographic axis, with *C*(6) graph set, through N1—H1A···O hydrogen bonds with D···A separations of 3.222 (2) Å (Fig. 2). There is also C12—H12···N3 non-classic hydrogen bonds with D···A distance of 3.379 (2) Å. The unit cell packing diagram of the title compound is shown in Fig. 3.

### Experimental

To a 1000 ml flask in ice bath, was added 6.36 g (0.05 mol) of *p*-chloroaniline and 4.68 g (0.13 mol) of HCl (d = 1.18 g.ml^-1^). To the obtained solution was added dropwise a solution of sodium nitrite (4.14 g in 25 ml H_2_O). Then, a diluted solution of *m*-anizidine (6.15 g, 0.05 mol) in 10 ml of methanol was added to the solution. The pH of the solution was adjusted at about 7–8 by adding a solution of 14.76 g of sodium acetate (0.18 mol) in 45 ml H_2_O as solvent. The solution was stirred for about 45 minutes, giving an orange precipitate. It was then filtered off and dried in vacuum. After dissolving in DMF and recrystallization, orange crystals of the title compound were obtained. M.p. 191–193 °C. Elemental Anal. calc. for C_13_H_12_ClN_3_O: C 59.66, H 4.62, N 16.06 %; ound: C 59.79, H 4.24, N 15.85 %. ^1^H-NMR (300 MHz, *d*_6_-DMSO) δ, ppm: 3.85 (3*H*, CH_3_), 6.19–7.69 (9*H*, aromatic and NH_2_ groups). ^13^C-NMR 100 MHz, DMSO) δ, ppm: 55.4 (O—CH_3_), 96.0–159.8 (C atoms of aromatic rings)

### Refinement

Methyl and aromatic H atoms were placed in idealized positions, with bond lengths fixed to 0.96 and 0.93 Å, respectively. Isotropic displacement parameters for these H atoms were calculated as *U*_iso_(H) = 1.5*U*_eq_(carrier C) in the case of the methyl group, and *U*_iso_(H) = 1.2*U*_eq_(carrier C) otherwise. Amine H atoms H1A and H1B were found in a difference map and refined isotropically, with free coordinates.

### Crystal data

**Table:**

C_13_H_12_ClN_3_O	*F*(000) = 1088
*M**_r_* = 261.71	*D*_x_ = 1.368 Mg m^−^^3^
Monoclinic, *C*2/*c*	Melting point: 464 K
Hall symbol: -C 2yc	Mo *K*α radiation, λ = 0.71073 Å
*a* = 15.398 (2) Å	Cell parameters from 9801 reflections
*b* = 12.132 (2) Å	θ = 2.4–31.8°
*c* = 14.276 (2) Å	µ = 0.29 mm^−^^1^
β = 107.65 (1)°	*T* = 293 K
*V* = 2541.3 (7) Å^3^	Irregular, red
*Z* = 8	0.30 × 0.17 × 0.11 mm

### Data collection

**Table:**

Bruker APEXII CCD diffractometer	2778 independent reflections
Radiation source: fine-focus sealed tube	2481 reflections with *I* > 2σ(*I*)
graphite	*R*_int_ = 0.019
φ and ω scans	θ_max_ = 27.0°, θ_min_ = 2.2°
Absorption correction: multi-scan (*SADABS*; Bruker, 2005)	*h* = −19→19
*T*_min_ = 0.698, *T*_max_ = 0.746	*k* = −15→15
42735 measured reflections	*l* = −18→18

### Refinement

**Table:**

Refinement on *F*^2^	Secondary atom site location: difference Fourier map
Least-squares matrix: full	Hydrogen site location: inferred from neighbouring sites
*R*[*F*^2^ > 2σ(*F*^2^)] = 0.039	H atoms treated by a mixture of independent and constrained refinement
*wR*(*F*^2^) = 0.118	*w* = 1/[σ^2^(*F*_o_^2^) + (0.063*P*)^2^ + 1.2073*P*] where *P* = (*F*_o_^2^ + 2*F*_c_^2^)/3
*S* = 1.04	(Δ/σ)_max_ < 0.001
2778 reflections	Δρ_max_ = 0.37 e Å^−^^3^
172 parameters	Δρ_min_ = −0.39 e Å^−^^3^
0 restraints	Extinction correction: *SHELXL97* (Sheldrick, 2008), Fc^*^=kFc[1+0.001xFc^2^λ^3^/sin(2θ)]^-1/4^
0 constraints	Extinction coefficient: 0.0021 (6)
Primary atom site location: structure-invariant direct methods	

### Fractional atomic coordinates and isotropic or equivalent isotropic displacement parameters (Å^2^)

**Table:**

	*x*	*y*	*z*	*U*_iso_*/*U*_eq_	
Cl	0.38256 (4)	0.12838 (5)	0.25280 (3)	0.0899 (2)	
N2	0.61207 (7)	0.40377 (9)	0.64610 (8)	0.0428 (3)	
O	0.72867 (7)	0.56731 (8)	0.66289 (7)	0.0538 (3)	
N3	0.56583 (8)	0.31792 (9)	0.64438 (8)	0.0450 (3)	
C8	0.70965 (9)	0.53992 (11)	0.74685 (9)	0.0425 (3)	
C1	0.52338 (9)	0.27653 (11)	0.54826 (9)	0.0435 (3)	
C7	0.64918 (9)	0.45106 (10)	0.73903 (9)	0.0401 (3)	
C9	0.74743 (10)	0.59254 (12)	0.83601 (10)	0.0478 (3)	
H9	0.7869	0.6517	0.8403	0.057*	
C11	0.66611 (10)	0.46804 (12)	0.91223 (10)	0.0485 (3)	
H11	0.6520	0.4436	0.9676	0.058*	
C10	0.72618 (10)	0.55670 (12)	0.91959 (10)	0.0484 (3)	
C12	0.62817 (9)	0.41745 (11)	0.82355 (10)	0.0437 (3)	
H12	0.5876	0.3595	0.8193	0.052*	
C6	0.51547 (11)	0.33586 (13)	0.46265 (11)	0.0543 (4)	
H6	0.5398	0.4065	0.4665	0.065*	
N1	0.76334 (13)	0.60746 (16)	1.00811 (11)	0.0723 (5)	
C2	0.48455 (12)	0.17279 (13)	0.54185 (12)	0.0590 (4)	
H2	0.4879	0.1338	0.5989	0.071*	
C3	0.44078 (12)	0.12672 (14)	0.45107 (13)	0.0658 (4)	
H3	0.4150	0.0569	0.4467	0.079*	
C4	0.43604 (11)	0.18557 (15)	0.36754 (12)	0.0589 (4)	
C5	0.47166 (12)	0.29024 (15)	0.37226 (11)	0.0619 (4)	
H5	0.4662	0.3298	0.3150	0.074*	
C13	0.78626 (14)	0.65978 (16)	0.66487 (13)	0.0711 (5)	
H13A	0.7942	0.6693	0.6012	0.107*	
H13B	0.8445	0.6475	0.7128	0.107*	
H13C	0.7589	0.7248	0.6820	0.107*	
H1A	0.7476 (16)	0.5869 (19)	1.0576 (17)	0.085 (7)*	
H1B	0.8002 (15)	0.6559 (18)	1.0126 (16)	0.075 (6)*	

### Atomic displacement parameters (Å^2^)

**Table:**

	*U*^11^	*U*^22^	*U*^33^	*U*^12^	*U*^13^	*U*^23^
Cl	0.0833 (4)	0.1190 (5)	0.0584 (3)	−0.0405 (3)	0.0080 (2)	−0.0312 (3)
N2	0.0442 (6)	0.0463 (6)	0.0377 (5)	−0.0031 (5)	0.0120 (4)	−0.0040 (4)
O	0.0662 (6)	0.0603 (6)	0.0355 (5)	−0.0191 (5)	0.0165 (4)	−0.0016 (4)
N3	0.0482 (6)	0.0454 (6)	0.0411 (6)	−0.0018 (5)	0.0130 (5)	−0.0032 (5)
C8	0.0470 (7)	0.0462 (7)	0.0343 (6)	−0.0015 (5)	0.0124 (5)	0.0004 (5)
C1	0.0411 (6)	0.0464 (7)	0.0425 (7)	−0.0017 (5)	0.0119 (5)	−0.0056 (5)
C7	0.0422 (6)	0.0428 (6)	0.0349 (6)	0.0011 (5)	0.0111 (5)	−0.0018 (5)
C9	0.0527 (7)	0.0489 (7)	0.0398 (7)	−0.0073 (6)	0.0111 (6)	−0.0038 (5)
C11	0.0585 (8)	0.0537 (7)	0.0369 (6)	0.0023 (6)	0.0197 (6)	0.0009 (5)
C10	0.0554 (8)	0.0524 (7)	0.0353 (6)	0.0022 (6)	0.0108 (6)	−0.0056 (5)
C12	0.0474 (7)	0.0444 (6)	0.0416 (6)	0.0002 (5)	0.0170 (5)	−0.0001 (5)
C6	0.0602 (8)	0.0547 (8)	0.0457 (7)	−0.0124 (7)	0.0126 (6)	−0.0032 (6)
N1	0.0930 (12)	0.0832 (11)	0.0395 (7)	−0.0252 (9)	0.0184 (7)	−0.0162 (7)
C2	0.0676 (9)	0.0545 (8)	0.0511 (8)	−0.0128 (7)	0.0121 (7)	0.0002 (6)
C3	0.0695 (10)	0.0576 (9)	0.0639 (10)	−0.0212 (8)	0.0105 (8)	−0.0104 (8)
C4	0.0496 (8)	0.0745 (10)	0.0481 (8)	−0.0131 (7)	0.0082 (6)	−0.0157 (7)
C5	0.0649 (9)	0.0738 (10)	0.0433 (7)	−0.0153 (8)	0.0110 (7)	−0.0027 (7)
C13	0.0866 (12)	0.0760 (11)	0.0548 (9)	−0.0334 (10)	0.0275 (9)	−0.0008 (8)

### Geometric parameters (Å, °)

**Table:**

Cl—C4	1.7391 (16)	C10—N1	1.3657 (19)
N2—N3	1.2578 (16)	C12—H12	0.9300
N2—C7	1.3985 (16)	C6—C5	1.378 (2)
O—C8	1.3586 (16)	C6—H6	0.9300
O—C13	1.4250 (18)	N1—H1B	0.81 (2)
N3—C1	1.4206 (17)	N1—H1A	0.85 (2)
C8—C9	1.3846 (18)	C2—C3	1.384 (2)
C8—C7	1.4065 (18)	C2—H2	0.9300
C1—C2	1.384 (2)	C3—C4	1.373 (2)
C1—C6	1.392 (2)	C3—H3	0.9300
C7—C12	1.4014 (18)	C4—C5	1.377 (2)
C9—C10	1.3987 (19)	C5—H5	0.9300
C9—H9	0.9300	C13—H13A	0.9600
C11—C12	1.3683 (19)	C13—H13B	0.9600
C11—C10	1.402 (2)	C13—H13C	0.9600
C11—H11	0.9300		
			
N3—N2—C7	115.10 (11)	C5—C6—C1	120.16 (14)
C8—O—C13	118.32 (11)	C5—C6—H6	119.9
N2—N3—C1	113.79 (11)	C1—C6—H6	119.9
O—C8—C9	123.74 (12)	C10—N1—H1B	119.4 (16)
O—C8—C7	115.59 (11)	C10—N1—H1A	119.5 (16)
C9—C8—C7	120.65 (12)	H1B—N1—H1A	121 (2)
C2—C1—C6	119.42 (13)	C3—C2—C1	120.45 (15)
C2—C1—N3	116.62 (13)	C3—C2—H2	119.8
C6—C1—N3	123.89 (12)	C1—C2—H2	119.8
N2—C7—C12	124.25 (12)	C4—C3—C2	119.06 (15)
N2—C7—C8	117.38 (11)	C4—C3—H3	120.5
C12—C7—C8	118.34 (11)	C2—C3—H3	120.5
C8—C9—C10	119.94 (13)	C3—C4—C5	121.45 (14)
C8—C9—H9	120.0	C3—C4—Cl	119.73 (13)
C10—C9—H9	120.0	C5—C4—Cl	118.81 (13)
C12—C11—C10	120.03 (12)	C4—C5—C6	119.40 (15)
C12—C11—H11	120.0	C4—C5—H5	120.3
C10—C11—H11	120.0	C6—C5—H5	120.3
N1—C10—C9	120.56 (15)	O—C13—H13A	109.5
N1—C10—C11	119.81 (14)	O—C13—H13B	109.5
C9—C10—C11	119.63 (12)	H13A—C13—H13B	109.5
C11—C12—C7	121.39 (13)	O—C13—H13C	109.5
C11—C12—H12	119.3	H13A—C13—H13C	109.5
C7—C12—H12	119.3	H13B—C13—H13C	109.5
			
C7—N2—N3—C1	175.50 (10)	C12—C11—C10—N1	179.51 (15)
C13—O—C8—C9	−4.1 (2)	C12—C11—C10—C9	−0.4 (2)
C13—O—C8—C7	177.23 (14)	C10—C11—C12—C7	1.1 (2)
N2—N3—C1—C2	169.37 (13)	N2—C7—C12—C11	−179.24 (12)
N2—N3—C1—C6	−13.7 (2)	C8—C7—C12—C11	−1.0 (2)
N3—N2—C7—C12	−8.56 (19)	C2—C1—C6—C5	−1.9 (2)
N3—N2—C7—C8	173.14 (11)	N3—C1—C6—C5	−178.75 (14)
O—C8—C7—N2	−2.78 (18)	C6—C1—C2—C3	2.1 (2)
C9—C8—C7—N2	178.49 (12)	N3—C1—C2—C3	179.20 (15)
O—C8—C7—C12	178.82 (12)	C1—C2—C3—C4	−0.3 (3)
C9—C8—C7—C12	0.1 (2)	C2—C3—C4—C5	−1.7 (3)
O—C8—C9—C10	−178.02 (13)	C2—C3—C4—Cl	179.22 (14)
C7—C8—C9—C10	0.6 (2)	C3—C4—C5—C6	2.0 (3)
C8—C9—C10—N1	179.64 (15)	Cl—C4—C5—C6	−178.99 (14)
C8—C9—C10—C11	−0.5 (2)	C1—C6—C5—C4	−0.1 (3)

### Hydrogen-bond geometry (Å, °)

**Table:**

*D*—H···*A*	*D*—H	H···*A*	*D*···*A*	*D*—H···*A*
N1—H1A···O^i^	0.85 (2)	2.47 (2)	3.222 (2)	147 (2)
C12—H12···N3^ii^	0.93	2.62	3.379 (2)	140

Symmetry codes: (i) *x*, −*y*+1, *z*+1/2; (ii) −*x*+1, *y*, −*z*+3/2.
